# A VLP-based vaccine provides complete protection against Nipah virus challenge following multiple-dose or single-dose vaccination schedules in a hamster model

**DOI:** 10.1038/s41541-017-0023-7

**Published:** 2017-08-08

**Authors:** Pramila Walpita, Yu Cong, Peter B. Jahrling, Oscar Rojas, Elena Postnikova, Shuiqing Yu, Lisa Johns, Michael. R. Holbrook

**Affiliations:** 1 School of Medicine, University of Hawaii, Manoa, Honolulu, HI USA; 2 National Institute of Allergy and Infectious Diseases, Integrated Research Facility, Fort Detrick, Frederick, MD 21702 USA

## Abstract

Nipah virus is a highly lethal zoonotic paramyxovirus that was first recognized in Malaysia during an outbreak in 1998. During this outbreak, Nipah virus infection caused a severe febrile neurological disease in humans who worked in close contact with infected pigs. The case fatality rate in humans was approximately 40%. Since 2001, NiV has re-emerged in Bangladesh and India where fruit bats (*Pteropus spp*.) have been identified as the principal reservoir of the virus. Transmission to humans is considered to be bat-to-human via food contaminated with bat saliva, or consumption of contaminated raw date palm sap, although human-to-human transmission of Nipah virus has also been documented. To date, there are no approved prophylactic options or treatment for NiV infection. In this study, we produced mammalian cell-derived native Nipah virus-like particles composed of Nipah virus G, F and M proteins for use as a novel Nipah virus vaccine. Previous studies demonstrated that the virus-like particles were structurally similar to authentic virus, functionally assembled and immunoreactive. In the studies reported here, purified Nipah virus-like particles were utilized either alone or with adjuvant to vaccinate golden Syrian hamsters with either three-dose or one-dose vaccination regimens followed by virus challenge. These studies found that Nipah virus-like particle immunization of hamsters induced significant neutralizing antibody titers and provided complete protection to all vaccinated animals following either single or three-dose vaccine schedules. These studies prove the feasibility of a virus-like particle-based vaccine for protection against Nipah virus infection.

## Nipah virus: Virus-like particles protect against major public health concern

A vaccine candidate consisting of virus-like particles could offer the first effective prophylaxis against the deadly Nipah virus. Endemic to Malaysia, Bangladesh, and India, Nipah virus is contracted from infected fruit bats and causes severe inflammation of the brain. Pramila Walpita, of the University of Hawaii, working with colleagues at the United States’ National Institute of Allergy and Infectious Diseases, developed an effective vaccine of immunity-generating Nipah virus surface proteins without the virus’ disease-causing genetic material. Their vaccine elicited a significant protective antibody response, with 100% of vaccinated animals surviving subsequent Nipah virus infection. Despite its prevalence and lethality, Nipah virus has no licensed vaccine and poses a serious problem to the developing countries it affects. Walpita’s vaccine candidate could offer a much-needed treatment option and warrants further investigation and pre-clinical trials.

## Introduction

Nipah virus (NiV) is a highly lethal zoonotic paramyxovirus. NiV infection can cause severe, rapidly progressive encephalitis in humans, with severe respiratory involvement in many cases. The first outbreak in Malaysia and Singapore in 1998 caused at least 107 deaths from a total of 265 known cases, resulting in a case fatality rate of ~40%.^1^ In the Malaysia outbreak, epidemiology pointed to pig farmers working in contact with pigs infected with NiV as the primary cause of the outbreak. The pigs had been infected, most likely through consumption of contaminated fruit or waste products from infected fruit bats (*Pteropus spp*.), the reservoir for NiV.^2–5^ Around 2001, NiV reemerged in Bangladesh and India causing smaller, but apparently deadlier outbreaks annually with case fatality rates between 70–100%.^6–8^ Transmission to humans in Bangladesh has been linked to consumption of food contaminated with bat saliva, or contaminated date palm sap.^4, 9, 10^ Person-to-person transmission in home or hospital settings has also been documented.^6–8, 11^ Importantly, NiV has potential as an agent of agro-terror since virus is transmitted in piggeries at a rate close to 100%.^3^ The high case fatality rate following NiV infection underscores the urgent need for prophylactic or therapeutic medical countermeasures.

NiV belongs to the family *Paramyxoviridae*, genus *Henipavirus*, which consists of NiV, Hendra (HeV) and Cedar (CedPV) viruses. NiV is a single-stranded RNA virus with six genes arranged consecutively, 3′-N, P, M, F, G and L-5′.^5^ The F (fusion) protein is produced as precursor F_0_ protein, a homotrimer, and is subsequently cleaved by cellular proteases into F_1_ and F_2_ subunits.^5^ F_1_ contains the viral fusion peptide that drives fusion between virus and host cell membranes.^5^ The L (large polymerase), P (phosphoprotein) and N (nucleocapsid) proteins are required for reconstructing the viral RNA polymerase activity, M (matrix) protein is required for morphogenesis and budding, and the G (glycoprotein) and F surface proteins are required for attachment and entry into the host cell.^12, 13^ In addition, the viral G protein, in particular, is required for stimulation of a neutralizing antibody response.^14, 15^

Several experimental vaccines have been evaluated for protection against NiV infection,^16^ including a canarypox virus-based,^17^ VSV∆G-based^18, 19^ and Rhabdovirus-based approaches.^20^ In addition, live attenuated vaccines and recombinant subunit G platforms have also been tested.^16^ Currently there is no licensed vaccine for treatment for NiV infection in humans. However, a HeV vaccine licensed in Australia for use in horses has shown cross-protection against NiV infection in non-human primates (NHP).^21^ In addition, a NiV-specific monoclonal antibody has been shown to be protective in NHP.^21^

Previously, we demonstrated production of mammalian cell-derived Nipah virus-like particles (NiV-VLPs) and validated their potential as a vaccine in Balb/c mice.^22^ The NiV-VLPs make highly effective immunogens because they possess features of authentic virus, including their surface structure and dimensions.^23–25^ NiV-VLPs are also safe because they do not contain viral genetic material or the ability to reconstitute the viral polymerase activity. We produced NiV-VLPs in mammalian cells to ensure structurally authentic mammalian N-glycosylation and O-glycosylation.^22^ These native VLPs, where one or more of the constituent proteins serve as immunogens, are particularly effective as vaccines for preventing disease following virus infection. VLPs can be produced safely, at scale and following appropriate regulatory requirements. Two VLP-based vaccines, Gardasil (Merck & Co) and Cervarix (GlaxoSimthKline), have been licensed and approved for use for prevention of human papillomaviruss infection. Two additional VLP-based vaccines, Sci-B-Vac (SciGen) and Bio-HepB (GlaxoSimthKline), have been licensed for prevention of Hepatitis B virus (HBV) infection. A number of additional VLP-based vaccines are currently in pre-clinical development or in clinical trials.^26–34^ The existence of licensed vaccines using the VLP platform validates the VLP-based approach for the development of a successful vaccine for preventing of NiV infection.

In the studies presented here, we expanded upon previous efforts that demonstrated functional assembly of NiV VLPs and immunogenicity in mice.^22^ To determine if NiV-VLPs induced protective immunity in a hamster challenge model for NiV infection, two approaches were used. The first approach used a three-dose vaccine schedule and the second used a single vaccination. In both trials, vaccinated animals developed significant neutralizing antibody titers following vaccination and demonstrated 100% protection against direct viral challenge. These data demonstrate that the NiV-VLP vaccine is protective against NiV infection and has significant potential for moving to further pre-clinical trials.

## Results

### Three-dose vaccination trial

**Fig. 1:**
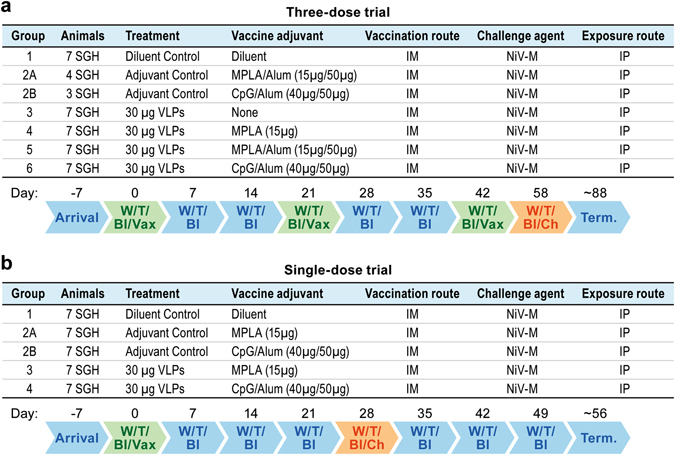
Study schedule for the two vaccine trials. Charts indicate the schedule for the **a** three-dose vaccine trial and **b** single-dose vaccine trial. Indicated are the activities on various days relative to vaccination. *W* Weight; *T* Temperature; *Bl* Bleed; *Vax* Vaccination; *Ch* Challenge; *Term* Termination

**Fig. 2:**
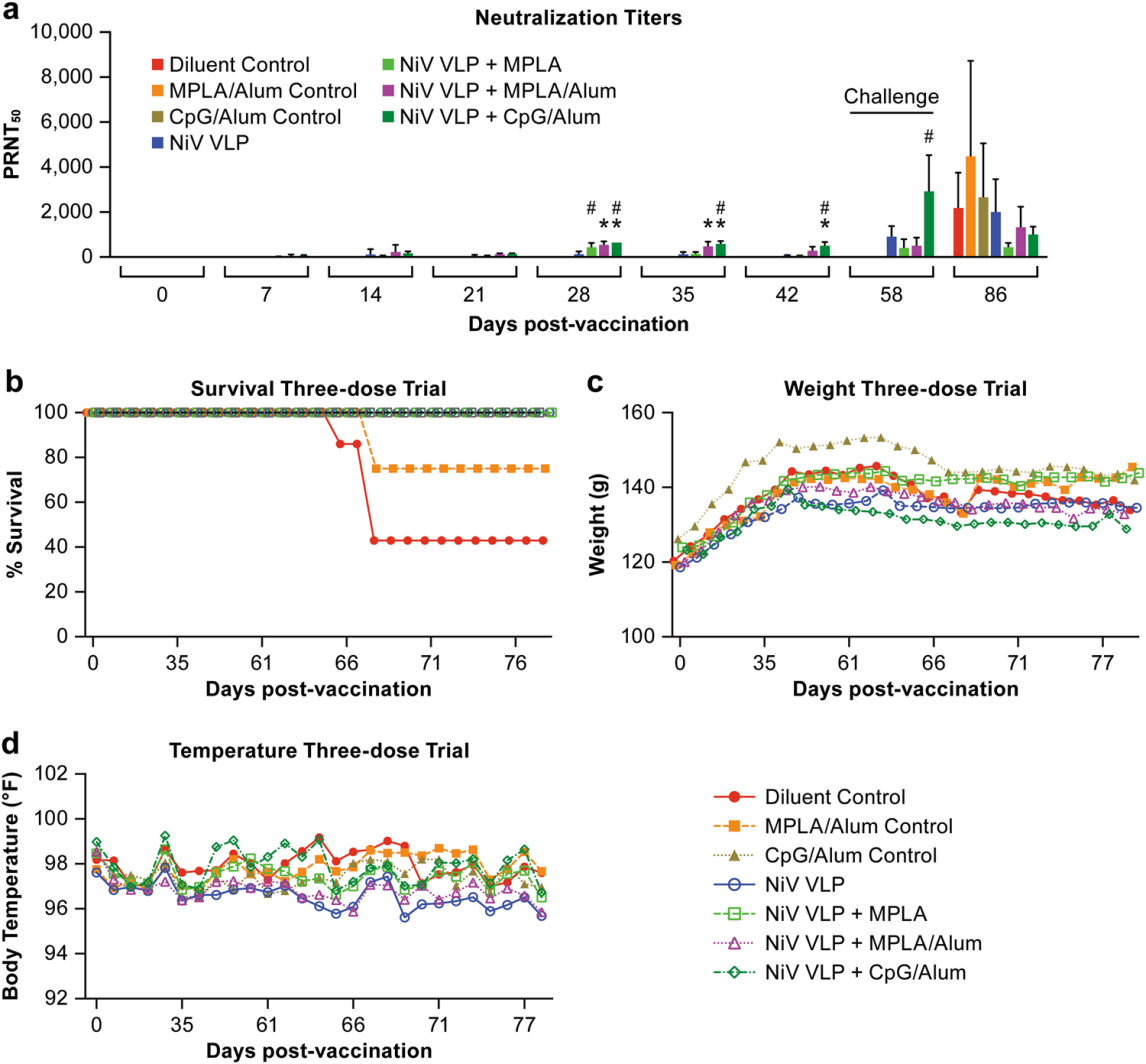
Neutralizing antibody titers and survival following challenge in a three-dose vaccination regimen. **a** Serum collected from vaccinated animals was tested for the presence of neutralizing antibodies following vaccinations. The (*) indicates a group average statistically (*P* < 0.01) greater than the diluent control for the same time point. The (#) indicates a group average statistically (*P* < 0.01) greater than the day 0 sample for the vaccination group, **b** Survival, **c** Weight change and **d** temperature change following vaccination and after viral challenge at day 58. In **b**, **c** and **d**, each *marker* represents the mean for the group

To determine efficacy of the NiV-VLPs as a vaccine, a three-dose vaccination schedule was followed in the hamster challenge model system. Using a vaccination schedule with doses delivered 3 weeks apart (days 0, 21 and 42) (Fig. 1a), neutralizing antibody titers were induced following vaccination in each of the vaccine groups, but not in diluent and adjuvant only controls. The groups using Alum as an adjuvant had notably higher neutralizing antibody titers than did those without Alum, with the titers increasing after each vaccination (Fig. 2a). Statistical analysis of the neutralization titers demonstrated significant (*P* < 0.01) differences between the vaccination groups and the diluent and adjuvant control groups beginning at 28 days post vaccination (Fig. 2a). Within individual vaccine groups significant (*P* < 0.01) differences were identified with the pre-vaccination sample by day 28 in the groups containing adjuvant.

Animals were challenged with a dose of 16,000 pfu of NiV via intraperitoneal (IP) inoculation on day 58 after initial vaccination. All vaccinated animals survived NiV challenge (Fig. 2b). None of the animals had marked changes in body weight or temperature over the course of the study (Fig 2c, d). As demonstrated in these studies, the adult hamster model for NiV infection is not a 100% lethal model as some animals can get ill, but not succumb to disease as is evidenced in the 43% survival rate in the mock vaccinated control group (Fig. 2b). The presence of adjuvants without the NiV VLP vaccine also provided a level of protection in these animals as the survival rate in the monophosphoryl lipid A (MPLA)/alum and CpG/Alum groups had 75 and 100% survival, respectively (Fig. 2b), despite all of the animals having overt signs of disease. Statistical analysis of the survival data demonstrated that survival in all three vaccination groups and the CpG/Alum control was significant (*P* < 0.05) relative to the diluent control group, but not the other two control groups (Fig. 2b).

#### Evaluation of virus dissemination by RT-PCR in the three-dose trial

**Fig. 3:**
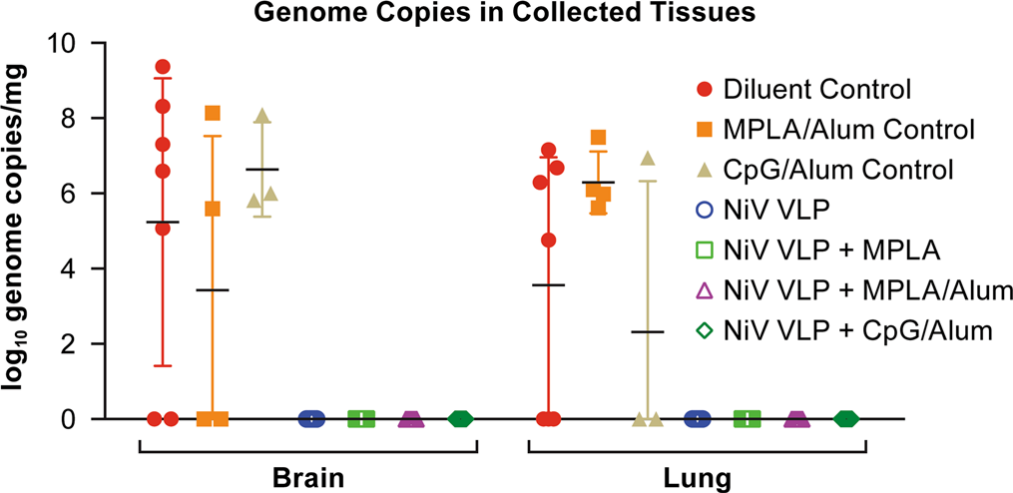
PCR data from the three-dose vaccine trial. Data indicate the log_10_ genome copies in the brain and lung collected at necropsy. Each *marker* represents an individual animal

To determine if animals had evidence of viral infection and replication, tissues collected at necropsy were assayed for the presence of viral RNA by qRT-PCR. Viral RNA was detected in the brain and/or lung of all but one animal in the control groups even though many of these animals survived infection (Fig. 3, Supplemental Table 1A). The one animal in which RNA was not detected survived the infection and also had minimal signs of disease. Viral RNA was not detected in tissues of any of the animals in the NiV-VLP-vaccinated groups. The presence of viral RNA in collected tissues in all but one of the control animals, and in none of the vaccinated animals, demonstrates that the control animals had a productive infection while the vaccinated animals did not.

### Single-dose vaccination trial

**Fig. 4:**
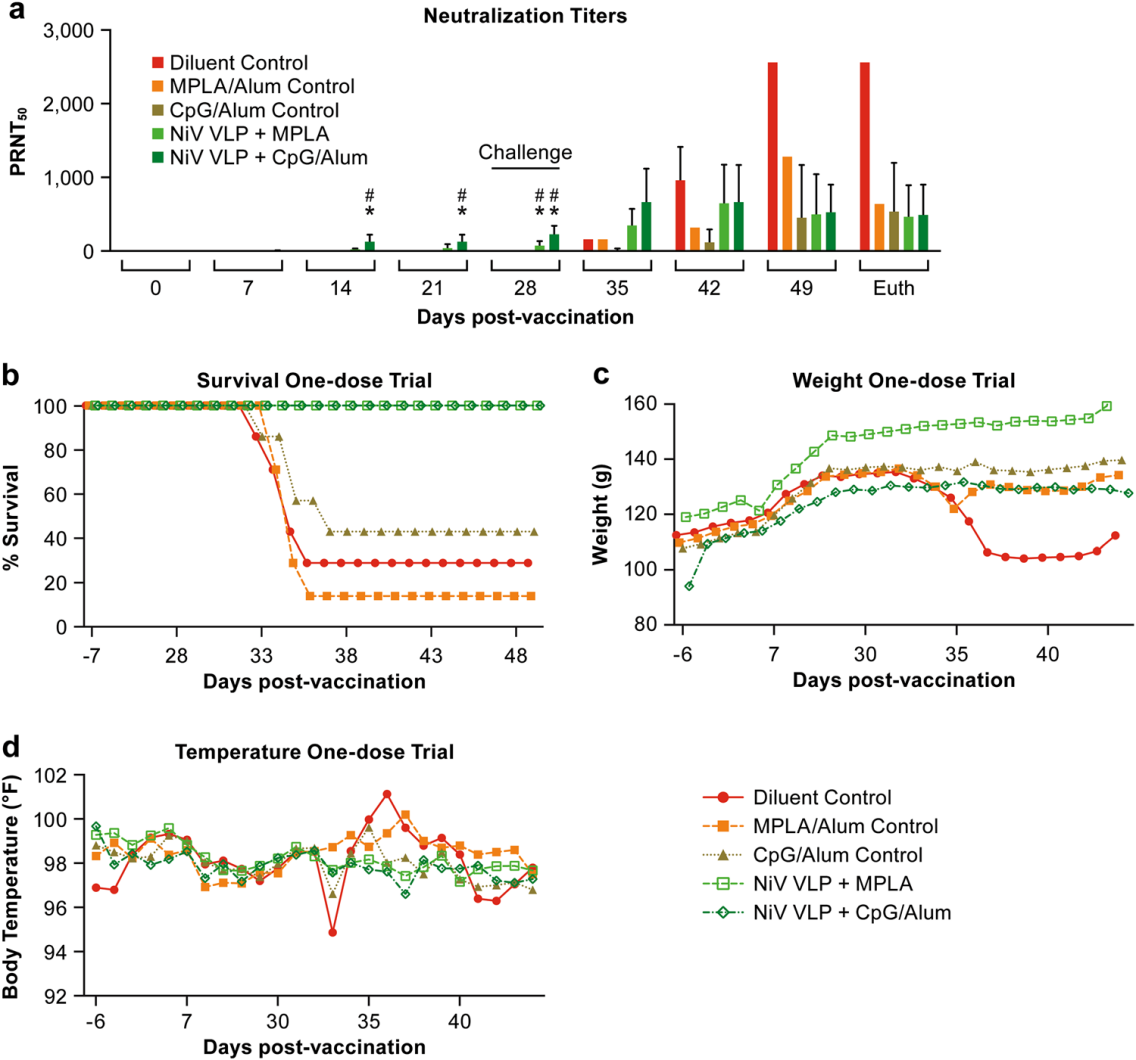
Neutralizing antibody titers and survival following challenge in a single-dose vaccination regimen. **a** Serum collected from vaccinated animals was tested for the presence of neutralizing antibodies following vaccinations. The (*) indicates a group average statistically (*P* < 0.01) greater than the diluent control for the same time point. The (#) indicates a group average statistically (*P* < 0.01) greater than the day 0 sample for the vaccination group, **b** Survival, **c** Weight change and **d** temperature change following vaccination and after viral challenge at day 28. In **b**, **c** and **d**, each *marker* represents the mean for the group

**Fig. 5:**
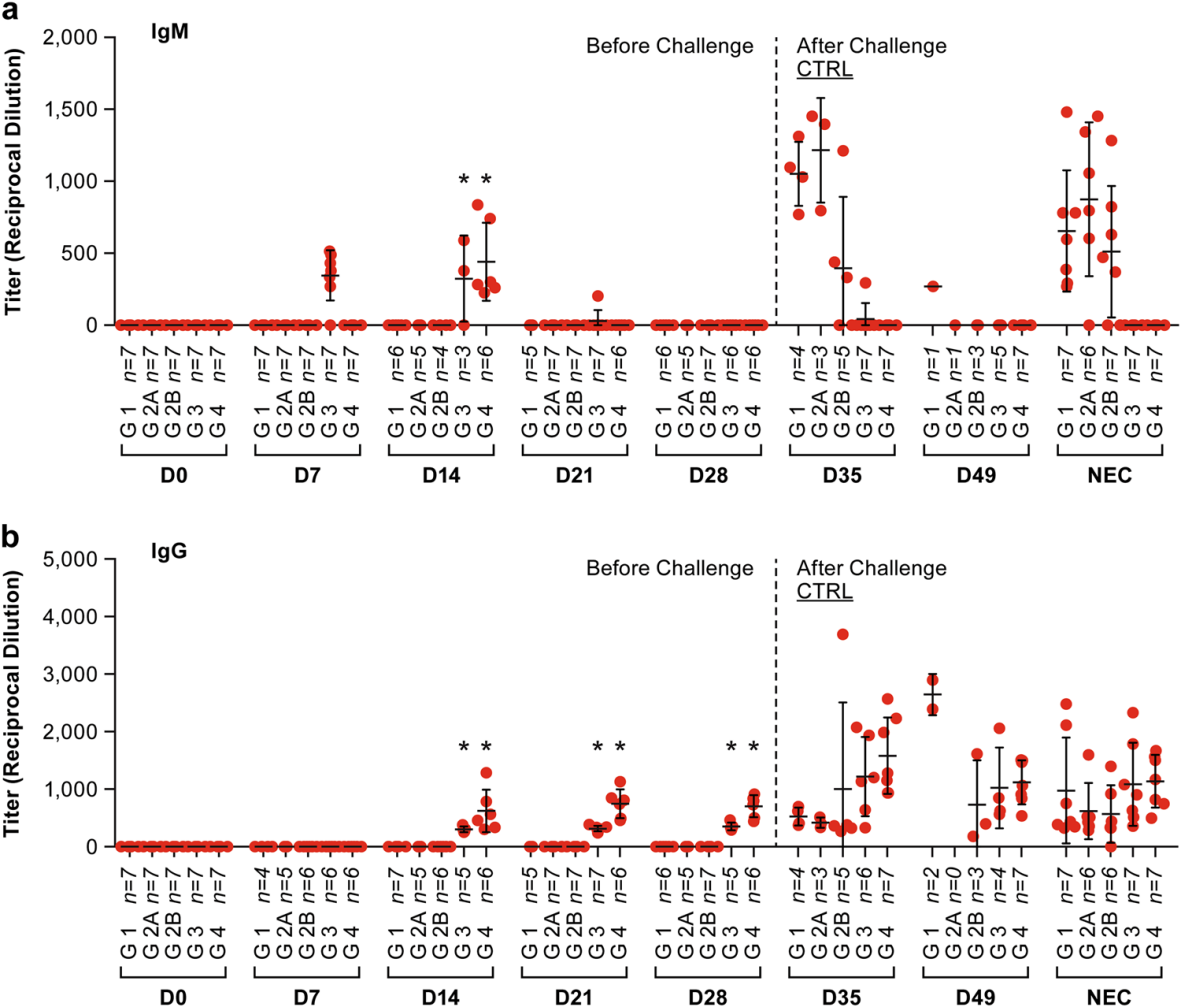
ELISA titers in serum collected during the single-dose vaccine trial. The *panel* **a** shows IgM titers and *panel* **b** shows IgG titers at the indicated days post-vaccination. Each marker represents an individual animal. Bars are the mean titer with standard deviation indicated. The (*) indicates a group average statistically (*P* < 0.01) greater than the diluent control for the same time point. The ELISA titers were determined based on the serum dilution that gives half-maximal response (EC50) of a 4-parameter logistic (4PL) sigmoidal dose-response (variable slope) curve. The endpoint of the graph was plotted with a 4PL nonlinear regression (curve fit) of corresponding percent maximal binding values in *Y*-axis (normalized from OD 450)^50^ versus the logarithm of the reciprocal serum dilution (*X*-axis) using GraphPad Prism software (version 7, GraphPad Software, La Jolla, CA)

As animals in the three-dose trial developed neutralizing antibody titers after a single NiV-VLP vaccine dose, an additional trial was performed to determine if a single vaccination was sufficient to protect animals against NiV challenge in the hamster model. In the initial trial the CpG/Alum and MPLA with or without Alum induced the most robust immune response 21 days after vaccination. Subsequently, in the single-dose trial, the CpG/Alum and MPLA adjuvants were tested in combination with the NiV-VLPs. Animals in the vaccination groups developed NiV-specific IgM titers progressing to IgG titers with a robust neutralizing antibody titer within 14 days post-vaccination (Figs. 4a, 5). There was no ELISA or neutralizing antibody titer in any of the control animals. The neutralization titers in the VLP + CpG/Alum group were significantly (*P* < 0.01) greater than the diluent control group by 14 days post-vaccination and greater than the adjuvant control groups by day 21 post-vaccination. The VLP + MPLA/Alum vaccination group only showed significant differences by day 28 post-vaccination. IgM titers in the vaccination groups were significantly different (*P* < 0.01) than all controls on day 14 post-vaccination only as the response waned by day 21 (Fig. 4a). The IgG titer in both vaccination groups was significantly greater (*P* < 0.01) than all controls on days 14, 21 and 28 post-vaccination (Fig. 4b).

Animals were challenged 28 days after vaccination with a more robust dose (33,000 pfu) of NiV compared to the three-dose trial in an effort to increase lethality in this model. Vaccinated animals had evidence of an anamnestic antibody response when tested 7 days after challenge while unvaccinated animals developed neutralizing antibody titers following challenge (Figs. 4a, 5). The increase in neutralizing antibodies in the control groups was likely due to the significant NiV-specific IgM response seen at 7 days post-challenge (35 days after vaccination) (Fig. 5a). All animals in the vaccinated groups survived infection with NiV while the majority of animals in the control groups succumbed to the infection (Fig. 4b). As was seen in the three-dose vaccine study, both CpG/Alum and MPLA adjuvants provided a level of protection in the absence of NiV-VLP. Three animals in the CpG/Alum control group (group 2B) survived NiV infection, but two of three showed clear signs of disease and had high neutralizing antibody titers following virus challenge. The third survivor in this group had low neutralizing antibody titers. In the MPLA control group (group 2 A) there was a single survivor that had minimal signs of disease and had a robust (1:640) neutralizing antibody titer at study termination. Statistical analysis demonstrated that survival in the MPLA + VLP vaccination group was significantly (*P* < 0.05) higher than all three of the control groups while survival in the CpG/Alum + VLP vaccination group was significantly higher than the diluent and MPLA control groups, but not the CpG/Alum control group (Fig. 4b).

#### Evaluation of virus dissemination by RT-PCR in the Single-dose trial

**Fig. 6:**
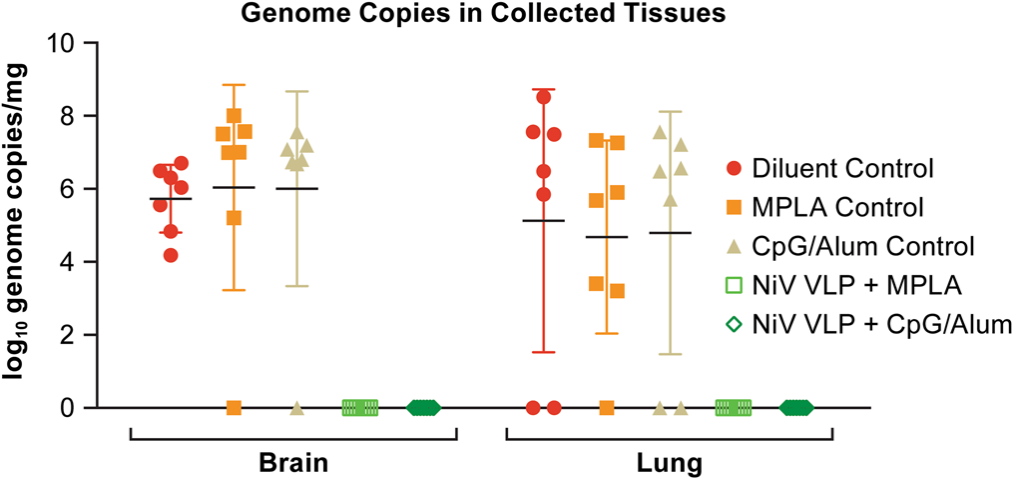
PCR data from the single-dose vaccine trial. Data indicate the log_10_ genome copies in the brain and lung collected at necropsy. Each *marker* represents an individual animal

In tissues collected at necropsy, viral genomic RNA was found in the brain and/or lungs of all but one control animal and in none of the vaccinated animals (Fig. 6, Supplemental Table 1B). The control animal without evidence of genomic RNA was in the CpG/Alum control group and survived the infection with no overt signs of disease. This animal was infected as it had a neutralizing antibody titer of 1:320 at study termination.

## Discussion

The generation of a safe and effective vaccine for the protection of humans from NiV infection is a critical public health priority in regions where NiV is endemic. The disease caused by NiV infection is highly lethal and potentially debilitating for survivors. In Australia there is a subunit vaccine licensed for protection of livestock, specifically horses, against infection by the closely related HeV.^21^ Although no licensed vaccine currently exists for protection against NiV infection, the Hendra vaccine was shown to be cross-protective against NiV infection in the NHP model .^35^ In the studies presented here, a non-replicating VLP-based vaccine was tested to determine if it stimulated immunity and protected hamsters against direct NiV challenge. These studies demonstrated that animals vaccinated with either a three-dose series or with a single NiV-VLP dose in the context of adjuvant developed a robust NiV-specific antibody response and were fully protected. These studies provide support for further development of a VLP-based vaccine for protection against NiV infection.

The use of VLP-based vaccines has been successfully tested for a number of viral systems.^26–29, 31–33^ In the studies presented here, we used NiV-VLPs expressing the viral M, F and G proteins that we have previously shown are structurally similar to authentic virus and express target viral proteins.^22^ Importantly, the viral F_0_ protein is cleaved during VLP processing and assembly to generate the biologically active proteins that are incorporated into the VLP. The presence of the virus F and G proteins in the VLP provide immunogenic targets for development of neutralizing antibodies.

Vaccine strategies involving VLPs are effective because they have the potential to produce both humoral and cell-mediated immune responses.^22, 24, 30^ As NiV-VLPs express the native viral F and G proteins, they bind and fuse with cell membranes in a manner similar to authentic virus, which allows for internalization of the VLPs and appropriate presentation of viral antigen in the context of MHC molecules. VLPs are also highly effective at generating protective antibody response because of their size range, particulate nature and their ordered and repetitive antigenic epitopes on the VLPs appears optimal for B cell activation.^22, 24, 30^

To determine if the NiV-VLPs could stimulate a protective immune response, hamsters were vaccinated with NiV-VLPs in the context of three different adjuvant formulations in a three-dose vaccine regimen. All vaccinated animals, including those vaccinated without adjuvant developed neutralizing antibodies after a single inoculation with robust titers after boost inoculations. Vaccinated animals were then challenged with NiV to determine if the induced antibody response was protective. All animals in the vaccinated groups survived and none showed significant signs of disease. Tissues collected from vaccinated animals had no evidence of NiV RNA, while there was significant evidence of viral RNA in either the brain or lungs of all but one control animal. These data demonstrate that vaccination with NiV-VLPs, even without adjuvant, could be effective for protection against NiV infection. However, a concern with the hamster model for NiV vaccine and therapeutic studies is that the infection is not 100% lethal in older (>~12 week-old) animals, despite animals showing significant signs of disease. That NiV infection is not uniformly lethal in older hamsters was not unexpected as the closely related HeV was not uniformly lethal in 11 week-old animals when challenged with (~1200 pfu) (100 LD_50_ as determined in 7 week-old hamsters) of virus.^36^ While uniform lethality in this model would be preferred, the combination of antibody titer in the vaccinated groups and significant signs of illness in unvaccinated animals provide a reasonable surrogate for protection in the absence of uniform lethality. Similar approaches are used for viruses such as dengue where non-lethal infection models are commonly used.^37^

In the three dose vaccine regimen, NiV-VLPs formulated with CpG and Alum induced neutralizing antibody titers of ~7.3log_2_ after the initial vaccination. This finding suggested that protection might be afforded after a single vaccination. To address this question, hamsters were vaccinated with a single NiV-VLP dose in the context of either MPLA or CpG/Alum adjuvants and then challenged with a high virus dose (33,000 pfu) at 28 days post vaccination. In this study, all of the vaccinated animals had NiV-specific IgM titers at 7 days post vaccination that transitioned to IgG titers beginning 14 days post vaccination. Following challenge, all vaccinated animals were completely protected and none showed signs of clinical disease. The younger age at the time of challenge (~13 weeks) and the increased challenge dose led to higher mortality in the control groups relative to the three-dose trial where the animals were ~17 weeks-old at the time of challenge. As with the three-dose study, the CpG/Alum adjuvant appeared to provide a level of protection in the absence of the NiV-VLP vaccine. Published studies have shown that CpG alone can provide protection against Venezuelan equine encephalitis,^38^ *Burkholderia pseudomallei* ^39^ and *Francisella tularensis* ^40^ infection in mouse models when provided several days prior to challenge. In addition, a recent study by Hamaoka et al identified a 20% survival rate in CpG only controls in a mouse challenge trial for development of a vaccine for *Pseudomonas aeruginosa* and using a similar vaccination schedule as was used in our three-dose vaccine trial.^41^ How CpG alone induces protective immunity against NiV infection several weeks after vaccination is unclear. CpG is a TLR-9 agonist that would enhance innate immunity, antibody responses and polarize cell-mediated immunity to a Th1 response, which is preferable for virus infections.^42^ While CpG induces various components of the innate immune response, the response should be dissipated by 21–28 days after stimulation.^43^

These studies demonstrate that the NiV-VLP vaccine has significant potential for additional development towards prophylactic use in humans. While the hamster model for NiV is not uniformly lethal, the development of significant neutralizing antibody titers in this small animal model provides a strong indicator of success, much as in similar studies with viruses that do not have a lethal immunocompetent animal model such as dengue and Zika viruses.^44–48^ The use of VLPs has a number of advantages over other vaccine platforms. Unlike live-attenuated or live-recombinant virus platforms, there is no risk of reversion or significant secondary effects that can be seen in recombinant platforms. Unlike subunit vaccines, VLPs can express multiple viral proteins that may be invaluable for inducing protective immunity. The VLPs also present viral surface proteins in their native state allowing the VLPs to stimulate production of appropriately reactive antibodies and to bind to and fuse with cells to introduce viral antigen to major histocompatibility complex (MHC) processing. VLPs can be produced at a reasonably large scale without the need for biocontainment laboratories. VLPs could also be lyophilized to potentially reduce the need for a cold chain in resource poor environments. While additional pre-clinical testing is necessary, the NiV-VLP platform holds promise as an effective prophylactic for the prevention of NiV infection in humans.

## Materials and methods

### Cells and virus

NiV-VLPs were generated in HEK 293 cells that had been adapted to suspension culture.^22^ The HEK 293 cells were maintained in FreeStyle 293 expression medium (ThermoFisher) at 37 °C/5% CO_2_.

The Malaysian strain of NiV was used in these studies. The virus was acquired from USAMRIID where the passage history documented three passages in VeroE6 and one passage in Vero cells. Following receipt at the NIAID-Integrated Research Facility (IRF), the challenge stock was passaged twice in VeroE6 cells (BEI #NR596). The viral genome of this stock was sequenced to ensure fidelity with the published genome sequence and the stock sequence uploaded to GenBank (IRF-160; KY425646.1). VeroE6 cells were maintained at the IRF in α-MEM w/GlutaMAX and incubated at 37 °C/5% CO_2_. All work with viable NiV was performed in the BSL-4 facility at the NIAID IRF in Frederick, MD.

### NiV-VLP production and purification

As previously described, NiV G, F and M protein genes were cloned into pCAGGS expression plasmids where gene expression is under control of a chicken β-actin promoter.^22, 49^ To generate VLPs, the plasmids were transfected into suspension adapted HEK 293 using Lipofectamine 2000 transfection reagent following manufacturer’s instructions (Invitrogen). VLPs were harvested from cell culture supernatant 48 h post-infection by centrifugation at 3500 rpm for 30 min at 4 °C to remove cell debris. Clarified supernatants were concentrated by ultracentrifugation through a 20% sucrose cushion in endotoxin free TN buffer (0.1 M NaCl; 0.05 M Tris-HCL, pH 7.4) at 27,000 rpm (Beckman SW28 rotor) for 2–4 h at 4 °C. The resulting NiV-VLP pellet was diluted in TN buffer, and purified on a discontinuous sucrose gradient formed by layering 65, 50, 20 and 10% sucrose in TN buffer. After centrifugation at 30,000 rpm (Beckman SW41 rotor) for 2 h, the NiV-VLP-containing band at the interface between the 20 and 50% sucrose layers was collected, diluted in TN buffer and concentrated by ultracentrifugation for 1 h through a 20% sucrose cushion. The resulting pellet of purified NiV-VLPs was re-suspended in a 5% sucrose solution in TN buffer and stored at 4 °C for subsequent analysis.

### VLP Protein concentration

The total protein concentration of the purified NiV-VLP preparations was measured by the BCA (Bicinchoninic acid) method following the manufacturer’s instructions (Thermo Scientific Laboratories).

### Animal studies

All animal studies were conducted in accordance with an Animal Study Protocol approved by the NIAID Division of Clinical Research (Protocol #IRF-022E) and University of Hawaii Animal Care and Use Committees and following recommendations in the Guide for the Care and Use of Laboratory Animals of the National Institutes of Health. Both institutions accept as mandatory the PHS policy on Humane Care of Vertebrate Animals used in testing, research and training. All animal work at NIAID is performed in facilities accredited by the American Association for the Accreditation of Laboratory Animal Care. Virus challenge was performed in the BSL-4 facility at the NIAID IRF.

For these studies, randomization of animals was not required due to the homogeneous nature of the groups. The investigators were not blinded as to the vaccination groups used in these studies.

### Challenge virus titration

Challenge virus titer was determined by back-titration in VeroE6 cells by plaque assay. The challenge virus stock was serially diluted 10-fold and inoculated onto multi-well plates containing 5 × 10^5^ VeroE6 cells/well seeded 2 days prior. The virus was allowed to adhere and infect for 1 h at 37 °C/5% CO_2_, rocking every 15 min. Following infection, cells were overlaid with semi-solid 1.25% Avicel (f/c) (FMC Biopolymer) diluted in Eagle's minimal essential medium followed by incubation for 3–4 days at 37 °C/5% CO_2_. The Avicel was then removed and the cells fixed and stained with neutral buffered formalin (NBF) containing 0.4% crystal violet (CV) (f/c) for 30 min at room temperature. After removing the CV staining buffer, the plates were washed with running water, and plaques enumerated.

### Vaccination and challenge

#### Vaccination with two booster dose trial

Golden Syrian hamsters (*Mesocricetus auratus*) (8–10 week-old, male) were purchased from Charles River Laboratories (CRL). Prior to study initiation, animals were implanted with temperature transponders (Bio Medic Data Systems) to record body temperature. Six groups of seven hamsters were used for the study (Fig. 1a). One group (group 2) was divided into two smaller adjuvant control groups. A total of three adjuvants were used in combination with the NiV-VLPs: Alum at 50 µg (Alhydrogel 2%); MPLA at 15 µg (MPLA-SM VacciGrade); and CpG at 40 µg (CpG ODN 1826, Class B), all from Invivogen. For all formulations that contained Alum, VLPs were mixed with Alum first, left at room temperature for 30 min, and then MPLA or CpG were added and mixed thoroughly. Animals were vaccinated via intramuscular (IM) inoculation in a volume of 100 µl.

Animals were bled (sublingual), vaccinated, weighed and their temperature recorded on days 0, 21 and 42 with post-vaccination bleeds on days 7, 14, 28 and 35. Animals were also bled immediately prior to challenge on day 58. Animals were challenged with ~16,000 pfu NiV-Malaysia by IP inoculation in a volume of 100 µl. Preliminary studies found the LD_50_ for ~10 week-old hamsters challenged by IP inoculation is less than 100 pfu, similar to what has previously been shown for HeV.^36^ Temperature and weight were recorded daily through 17 days post challenge when the critical phase of disease had passed. Afflicted animals were euthanized when they had significant signs of disease. Surviving animals were euthanized at the end of the study, approximately day 88 post vaccination. One half of the brain and the left lung lobe were collected from all animals at necropsy for polymerase chain reaction (PCR) analysis.

#### Single vaccination trial

Hamsters (*M. auratus*) (8–10 week-old, male) were purchased from CRL and were implanted with temperature transponders prior to study initiation. Five groups of seven hamsters were used for the study (Fig. 1b). Three control groups including a diluent control (Group 1) and two adjuvant control (Groups 2 A and 2B) groups were included. Two adjuvants were used in combination with the NiV-VLPs: MPLA at 15 µg (Group 3); and CpG at 40 µg mixed with 50 µg Alum (Group 4). For the Group 4 formulation that contained Alum, VLPs were mixed with Alum first, left at room temperature for 30 min, and then CpG was added and mixed thoroughly. The vaccines were loaded into individual syringes. Animals were vaccinated via IM inoculation in a volume of 100 µl.

Animals were weighed and their temperature recorded several days prior to vaccination and on days 0, 7, 14 and 21. Animals were bled and vaccinated on day 0. Post-vaccination bleeds were collected on days 7, 14, 28, 35, 42, 49 and at necropsy. On day 28 post vaccination, animals were challenged with ~ 33,000 pfu NiV-Malaysia by IP inoculation in a volume of 100 µl. Temperature and weight were recorded daily through 14 days post challenge (day 42 post-vaccination) when the critical phase of disease had passed. Animals showing significant signs of disease were euthanized following approved protocols. Surviving animals were euthanized at the end of the study, approximately day 56 post vaccination. One half of the brain and the left lung lobe were collected from all animals at necropsy for PCR analysis.

### ELISAs

#### Nipah antigen preparation

NiV antigens for ELISAs were obtained from cell culture supernatant and crude extracts of VeroE6 cells inoculated with NiV. Cell culture supernatant was collected, clarified by centrifugation, aliquoted and stored at −80 °C. Cells were harvested and washed with phosphate-buffered saline (PBS), then lysed in radioimmunoprecipitation assay buffer (Cell Signaling) with protease inhibitors (Roche). The lysate was incubated at 4 °C for 10–20 min before freezing at −80 °C. Supernatants and cell extracts were irradiated (5 Mrad) to inactivate viable virus prior to use. The irradiated cell lysate was sonicated four times (30 s each) and clarified by centrifugation at 10,000 rpm, 4 °C for 15 min. The cell extract was aliquoted and frozen at −80 °C. The supernatant was used as the source of NiV antigen for IgM ELISAs and the cell extract was used as the antigen in IgG ELISAs.

NiV GP-specific rabbit polyclonal antiserum used in the IgM ELISA was generated by Thermo Scientific using NiV GP “293 FreeStyle Tet-NiV-sG” (a gift from Dr. Christopher C. Broder) as an antigen.

#### Indirect NiV IgM ELISA (IgM Capture ELISA)

Mouse-anti-Armenian Hamster IgM antibody (BD Pharmingen #554031) was diluted to a concentration of 0.2 µg/100 µl/well in cold PBS and adsorbed to 96-well ELISA plates (Thermo Scientific) at 4 °C overnight. After discarding the coating solution, plates were washed with phosphate-buffered saline with Tween 20 (PBST) six times and incubated at 37 °C for 2 h with blocking solution (3% chicken serum/2% milk in PBST). Heat inactivated test sera was added in two-fold serial dilutions (40 µl/well) in blocking solution containing normal hamster serum at 1:400 and incubated at 4 °C overnight. Plates were washed six times with PBST. NiV antigen was added to the plates (0.5x10^5^ pfu/well in 50 µl), followed by incubation at 37 °C for 1 h. Plates were washed six times with PBST before NiV-specific polyclonal antibody was applied at 1:4000 dilution and samples incubated at 37 °C for 1 h. The plates were washed six times with PBST. Goat-anti-rabbit IgG (whole molecule)-HRP (Sigma #6154) diluted 1:10,000 in blocking solution was added to the plates and incubated at 37 °C for 1 h. Following six washes with PBST, tetramethylbenzidine (TMB) substrate (Thermo Scientific) was added and the plates incubated at room temperature for 5 min. The reaction was terminated by addition of stop solution (Thermo Scientific). The ODs were measured at 450 nm on an Infinite M1000 TECAN plate reader. Reciprocal serum dilutions corresponding to 50% maximal binding were used to calculate the titers.

#### Direct NiV IgG ELISA

Inactivated cell extracts were diluted at 1:5000 (final protein concentration 0.05 µg/well) with carbonate buffer, pH 9.5 (Biolegend), coated on plates in a volume of 50 µl and incubated overnight at 4 °C. The plates were washed with PBST six times before 300 µl blocking buffer was added and incubated at 37 °C for 2 h. Two-fold serial dilutions of heat-inactivated test sera were added to the plates and incubated at 4 °C overnight. Blocking solution with normal hamster serum added at 1:400 was used as the serum diluent. Plates were washed six times with PBST before adding goat anti-Syrian Hamster IgG (H+L)-HRP (Abcam ab6892) diluted 1:10,000 in blocking solution and incubated at 37 °C for 1 h. Plates were washed six times with PBST and the samples developed using TMB substrate (room temperature for 5 min). The reaction was stopped by addition of stop solution and the OD measured at 450 nm on an Infinite M1000 TECAN plate reader. Reciprocal serum dilutions corresponding to 50% maximal binding were used to calculate the titers.

### Neutralization assays

Antibody neutralization titers were determined by either fluorescence reduction neutralization assays (FRNA) (three-dose trial) or tissue culture infectious dose (TCID) (single-dose trial) reduction assays. Prior to using the TCID neutralization assay in the single vaccination study, the TCID and FRNA assays were compared through correlation analyses using samples collected at two different days post-vaccination. The assays were found to be equivocal (*R* ^*2*^ = 0.7–0.8). For both assays, test sera were heat inactivated prior to testing, serially diluted two-fold and incubated with a fixed concentration of NiV (multiplicity of infection = 0.1) for 1 h at 37 °C/5% CO_2_. For FRNAs, triplicate samples of the virus-antibody mixtures were added to monolayers of VeroE6 cells and incubated for 2 days at 37 °C/5% CO_2_. The cells were then fixed with NBF for 30 min at room temperature. After fixation, cells were washed three times with PBS, permeabilized with 0.25% Triton X-100 in PBS for 5 min at room temperature and washed again three times with PBS. Cells were blocked with 10% normal goat serum (NGS) in PBS for 1 h at room temperature. Cells were then incubated at 4  °C overnight with a NiV G protein-specific rabbit polyclonal antiserum diluted 1:2000 in 1.5% NGS in PBS. After incubation, the cells were washed three times with PBS and incubated with goat anti-rabbit IgG (H+L) secondary antibody, Alexa Fluor^®^ 488 conjugate (Life Technologies) for 30 min at room temperature. The cells were washed and positive wells quantified by a Tecan fluorescence plate reader and confirmed by microscopy.

For TCID reduction assays, heat inactivated hamster serum or mock serum samples were serially diluted two-fold in duplicate in 96-well plates. NiV was added to each well, except uninfected controls, at a concentration of 500 pfu/well and incubated for 1 h at 37 °C. The serum/NIV mixture was then transferred to 96-well plates seeded a day prior with 4 × 10^5^/well VeroE6 cells. The plates were incubated 5 days at 37 °C/5%CO_2_. Following incubation, plates were fixed and stained with 0.25% CV in NBF. The neutralizing antibody titer was determined based on the antibody dilution endpoint at the onset of significant cytopathic effects in duplicate wells.

### Statistics

The group size required to provide sufficient power in these vaccine trials was determined to be seven animals. The comparison between treatment and control provides a calculation for a one-sided test with type one error of 0.05. For this determination, it was reasonable to assume that the treatment (in terms of survival) is at least as good as the control. Power was calculated to provide a value greater than 84% for survival in treatment groups was greater than 0.7 when survival in the controls was assumed to be 0.

In order to determine statistical significance for survival in the two vaccine studies, Kaplan–Meier survival data were compared using Mantel-Cox logrank tests and significance was defined as *P* < 0.05. For neutralization assays significance was determined on individual days post-vaccination and between days within individual vaccination groups using a two-way ANOVA following Tukey’s multiple comparison test. Significance was defined as *P* < 0.01. For ELISAs, significance was determined between groups over time and within vaccination groups using a standard two-way ANOVA with significance defined as *P* < 0.01. All statistical analyses were performed using Prism 7.0 (GraphPad).

### Data availability statement

All relevant data from this study are available from the authors.

## Electronic supplementary material

Supplemental Table 1
