# Supplementary material for: A VLP-based vaccine provides complete protection against Nipah virus challenge following multiple-dose or single-dose vaccination schedules in a hamster model
Source: NPJ Vaccines. 2017 Aug 8;2:21. doi: 10.1038/s41541-017-0023-7 (PMC5627259; doi:10.1038/s41541-017-0023-7)
Supplement: Supplementary file 1 — Supplemental Table 1 [file 41541_2017_23_MOESM1_ESM.docx]

|  | Diluent Control | | MPLA/Alum Control | | CpG/Alum Control | | VLPs | | VLPs + MPLA | | VLPs + MPLA/Alum | | VLPs+ CpG/Alum | |
| --- | --- | --- | --- | --- | --- | --- | --- | --- | --- | --- | --- | --- | --- | --- |
| Animal ID | Brain | Lung | Brain | Lung | Brain | Lung | Brain | Lung | Brain | Lung | Brain | Lung | Brain | Lung |
| 1 | 7.3 | 0 | 0 | 6.1 | 8.09 | 6.95 | 0 | 0 | 0 | 0 | 0 | 0 | 0 | 0 |
| 2 | 9.37 | 6.68 | 8.14 | 7.49 | 5.82 | 0 | 0 | 0 | 0 | 0 | 0 | 0 | 0 | 0 |
| 3 | 5.07 | 6.29 | 5.59 | 5.62 | 6.01 | 0 | 0 | 0 | 0 | 0 | 0 | 0 | 0 | 0 |
| 4 | 0 | 0 | 0 | 5.98 |  |  | 0 | 0 | 0 | 0 | 0 | 0 | 0 | 0 |
| 5 | 8.32 | 7.16 |  |  |  |  | 0 | 0 | 0 | 0 | 0 | 0 | 0 | 0 |
| 6 | 6.59 | 0 |  |  |  |  | 0 | 0 | 0 | 0 | 0 | 0 | 0 | 0 |
| 7 | 0 | 4.76 |  |  |  |  | 0 | 0 | 0 | 0 | 0 | 0 | 0 | 0 |

**Supplemental Table 1. Lung and brain virus titers from individual animals in the vaccine trials**

**a.**

**b.**

|  | Diluent Control | | MPLA Control | | CpG/Alum Control | | VLPs + MPLA | | VLPs+ CpG/Alum | |
| --- | --- | --- | --- | --- | --- | --- | --- | --- | --- | --- |
| Animal ID | Brain | Lung | Brain | Lung | Brain | Lung | Brain | Lung | Brain | Lung |
| 1 | 4.84 | 6.48 | 6.99 | 0 | 7.56 | 7.22 | 0 | 0 | 0 | 0 |
| 2 | 6.04 | 8.52 | 0 | 3.4 | 6.81 | 6.57 | 0 | 0 | 0 | 0 |
| 3 | 6.49 | 0 | 5.2 | 3.2 | 6.68 | 6.48 | 0 | 0 | 0 | 0 |
| 4 | 6.7 | 7.49 | 7.5 | 5.68 | 7.08 | 7.56 | 0 | 0 | 0 | 0 |
| 5 | 5.56 | 7.56 | 7 | 5.91 | 7.19 | 5.72 | 0 | 0 | 0 | 0 |
| 6 | 6.3 | 0 | 7.57 | 7.26 | 6.74 | 0 | 0 | 0 | 0 | 0 |
| 7 | 4.18 | 5.85 | 8.01 | 7.33 | 0 | 0 | 0 | 0 | 0 | 0 |
